# Trends in the incidence of asthma, atopic dermatitis, and multiple sclerosis before, during, and after the COVID-19 pandemic in a US claims database

**DOI:** 10.1371/journal.pone.0355103

**Published:** 2026-07-30

**Authors:** Kexin Zhu, Julie Barberio, Nicole Tsao, Anil Mor, Venkatesh Harikrishnan, Xinyu Li, Sarah-Jo Sinnott

**Affiliations:** 1 Epidemiology and Benefit Risk, Sanofi, Morristown, New Jersey, United States of America; 2 Epidemiology and Benefit Risk, Sanofi, Cambridge, Massachusetts, United States of America; 3 Global Medical Affairs, Sanofi, Cambridge, Massachusetts, United States of America; Karolinska Institutet, SWEDEN

## Abstract

There is limited evidence on how reduced healthcare resource utilization during the COVID-19 pandemic has affected the detection of inflammatory and immunologic diseases. We aimed to describe the observed incidence rates (IRs) of asthma, atopic dermatitis (AD), and multiple sclerosis (MS) before, during, and after the pandemic. Individuals aged ≥6 years were identified in Optum’s de-identified Clinformatics^®^ Data Mart Database from 2018 to 2022 to make 20 season-based cohorts. Age- and sex-standardized IRs of asthma, AD, and MS were estimated. Incidence rate ratios (IRR) and 95% confidence intervals (CI) were calculated comparing IRs in seasonal cohorts in 2019–2022 to the corresponding timeframe in 2018. Compared to spring 2018, IRs of asthma, AD, and MS in spring 2020 decreased by 14% (IRR: 0.86, 95% CI: 0.84–0.87), 28% (IRR: 0.72, 95% CI: 0.69–0.75), and 23% (IRR: 0.77, 95% CI: 0.68–0.87), respectively. The observed incidence reduction was most profound in children (6–11 years) and adolescents (12–17 years), followed by senior adults (≥65 years). There was no sex difference. IRs returned to or exceeded pre-pandemic levels for AD and MS in summer 2020 and for asthma in spring 2021. COVID-19 led to an apparent decline in incidence for selected inflammatory and immunologic diseases, which was more pronounced for pediatric and senior populations. The observed decrease in incidence likely reflects delayed access to healthcare, resulting in unrecorded (but still occurring) diagnoses for those time periods. Future studies using data that encompass the pandemic period should exercise caution in the design of study and interpretation of incidence or prevalence data.

## Introduction

During the early period of the COVID-19 pandemic, healthcare facilities restricted or postponed their services for elective, non-essential, and non-urgent purposes [1–3]. In addition, many patients avoided seeking both urgent and routine care due to concerns about contracting COVID-19 [4]. Prior research has demonstrated significant declines in access to routine medical care, decreases in referrals, and reduced inpatient admissions across the United States (US) among patients without COVID-19 in the early stage of the pandemic (e.g., winter and spring of 2020) [5–9].

Reduced healthcare utilization during the pandemic led to lower detection and reporting of non-COVID-19 related diseases [10,11], which likely translated to an artificially reduced incidence during the pandemic compared to pre-pandemic and post-pandemic timepoints. While the recorded diagnosis of cancer and cardiovascular outcomes from March to May 2020 compared to pre-pandemic has been well described in the literature [12,13], limited research has been conducted on the changes in the recorded new diagnoses of inflammatory and immunologic diseases. Furthermore, it is known that young children and older adults were disproportionately impacted by health service restrictions during the pandemic [4,7]. However, trends in the recorded new diagnoses of diseases by age or sex, as impacted by COVID-19 and its related restrictions, have not been well-researched. Lastly, existing literature on healthcare utilization and change in incidence have been primarily based on data in the first pandemic year [2,7,9,12], and evidence is limited on the impacts of the pandemic (e.g., recovery of healthcare utilization, other COVID-19 related changes) on estimates of disease incidence in the long term.

In this study, we estimated the recorded incidence rates (IRs) of asthma, atopic dermatitis (AD), and multiple sclerosis (MS) in multiple season-based cohorts from 2018 through 2022 in a claims database in the US. We compared IRs in each cohort to those in the same seasonal cohort in 2018 (baseline reference). Furthermore, IRs were calculated by age and sex to identify whether estimates of recorded incidence were disproportionately affected in one group more than another. This descriptive study was focused on the methodological implications of the pandemic for incidence estimation using a US claims database, rather than on causal inferences about COVID-19 or specific countermeasures and disease risk.

## Materials and methods

### Data source and study population

This was a cohort study using Optum’s de-identified Clinformatics^®^ Data Mart Database (CDM) from December 2016 through November 2023. The Optum^®^ CDM includes administrative health claims for commercial insurance and Medicare Advantage health plans from enrollees in all 50 US states.

Previous studies have observed the seasonal variations in healthcare services utilization related to asthma, AD, and MS [14–16]. To account for seasonality, IRs of asthma, AD, and MS were calculated in cohorts based on seasons from winter 2018 (December 1, 2017 to February 28, 2018) to fall 2022 (September 1, 2022 to November 30, 2022); therefore, 20 seasonal cohorts for each outcome of interest were created. For each seasonal cohort, individuals eligible for inclusion met the following criteria: (1) were enrolled in the Optum^®^ CDM for at least one day in the season, (2) had 12 months of continuous enrollment prior to the index date, (3) aged at least 6 years old, (4) had no missing or unknown sex information, and (5) had no evidence of the outcomes of interest (defined below) within 12 months before the index date. The index date was the earliest date that eligibility criteria were met during the season.

### Outcome measures

For asthma and AD, incident case was identified by ≥2 inpatient or outpatient International Classification of Diseases, Tenth Revision, Clinical Modification (ICD-10-CM) diagnostic codes for asthma (J45) or AD (L20.0, L20.81, L20.82, L20.84, L20.89, L20.9) that were 30–365 days apart. The date of the outcome occurrence was the date of the first qualifying ICD-10-CM code for asthma or AD identification. For incident MS cases, patients were required to have ≥2 inpatient or outpatient ICD-10-CM diagnostic codes for MS (G35) (30–365 days apart) and ≥1 dispensing for disease modifying therapies (DMT) (S1 Table) within one year after the first qualifying ICD-10-CM code for MS identification (positive predictive value [PPV] of 95.4%–97.8% and sensitivity of 87.2%–93.4% [17]). The date of the first qualifying ICD-10-CM code for MS was defined as the date of the outcome occurrence. For all three outcomes, the date of the first qualifying code was required to fall in the cohort timeframe (within each season), while the second ICD-10-CM code and the date of dispensation for DMT could go beyond the cohort timeframe but should be within one year of the first qualifying ICD-10-CM code.

### Follow-up

For each seasonal cohort, each individual was followed from the index date until the date of the outcome of interest (defined above), death, disenrollment of health insurance coverage, or the end of the cohort timeframe (last date of the season), whichever came first.

### Statistical analysis

The incidence rate (IR) was calculated as the number of individuals with an incident outcome divided by the sum of each individual’s person-time at risk (from the index date to the end of follow-up in the unit of person-years) in each seasonal cohort from 2018 to 2022. The corresponding 95% confidence intervals (CI) were calculated using an Exact method [18]. Incidence rate ratios (IRR) and 95% CIs comparing the IRs in each seasonal cohort in 2019–2022 to the IRs in the corresponding timeframe in 2018 (baseline reference) were calculated using Poisson regression with the Exact method [18]. Age- and sex-standardized IRs and IRRs were calculated using a direct method with the 2022 US population composition by age and sex from the US Census Bureau as the reference population to reflect the most current population structure at the end of our study period [19].

In subgroup analyses, IRs were calculated by age (6–11, 12–17, 18–64, and ≥65 years) and sex (male, female) subgroups in each seasonal cohort. IRRs were calculated to compare the IRs in each age and sex subgroup in 2019–2022 to the IRs in the corresponding age and sex subgroups in the 2018 seasonal cohort as a reference. All statistical analyses were performed in SAS version 9.4 (SAS Institute, Cary, NC, USA).

### Ethical approval

This study involved only secondary database analysis of de-identified administrative health claims data and is not considered human subject research. No institutional review board approval was required.

## Results

S2 Table presents the characteristics of the study population included in the seasonal cohorts for each outcome in 2018. Each seasonal cohort had slightly different study sample sizes and characteristics due to different outcomes under study and eligibility criteria at cohort entry. Approximately 11 million individuals were included in each cohort with an average age of 49–51 years and equal sex distributions (51% females).

**Fig 1:**
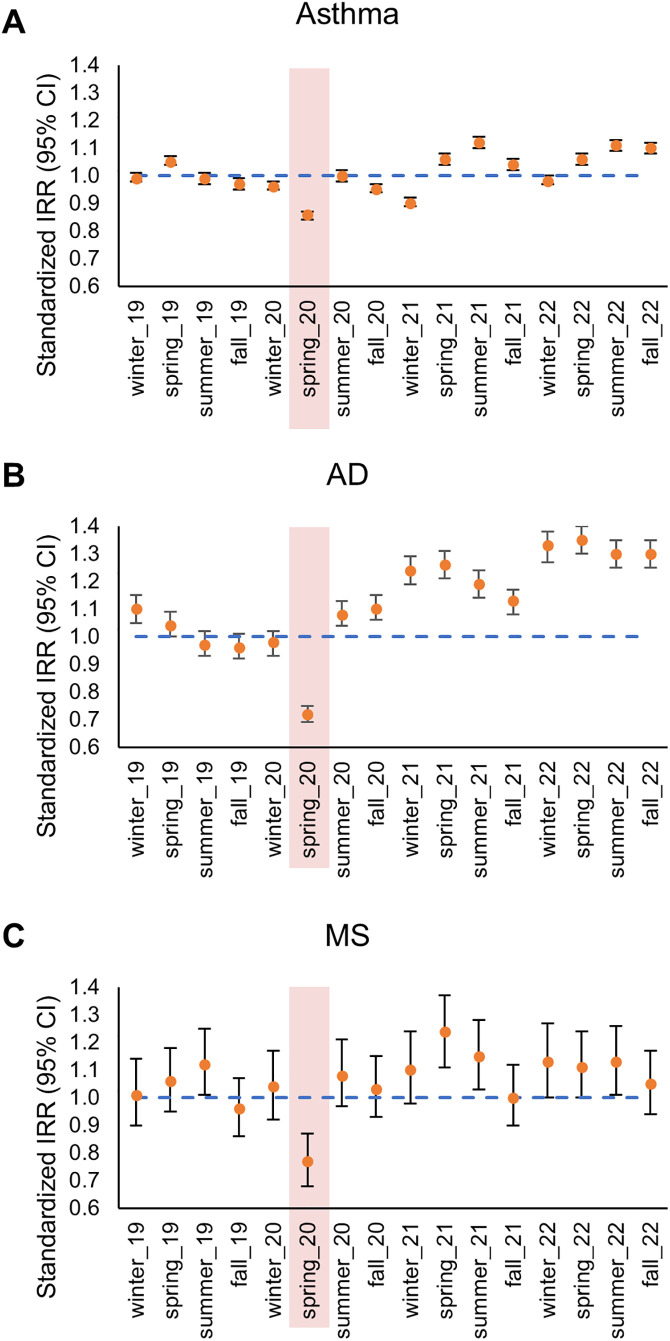
Incidence rate ratios (IRR) of asthma, atopic dermatitis (AD), and multiple sclerosis (MS) in seasonal cohorts from 2018 to 2022 in Optum^®^ CDM. Dots represent IRRs and error bars represent the 95% confidence intervals. Dash lines represent an IRR of 1. IRRs were calculated to compare the incidence rates (IRs) in each seasonal cohorts in 2019-2022 to the IRs in the corresponding timeframe in 2018 (baseline reference). Standardized IRs were calculated using a direct method with the population composition by age and sex in the United States in 2022 as the reference population.

S1 Fig presents IRs of asthma, AD, and MS in each seasonal cohort from 2018 to 2022. IRs for all three outcomes declined during spring 2020. Compared to spring 2018, IRs of asthma, AD, and MS decreased by 14% (IRR: 0.86, 95% CI: 0.84, 0.87), 28% (IRR: 0.72, 95% CI: 0.69, 0.75), and 23% (IRR: 0.77, 95% CI: 0.68, 0.87), respectively, during spring 2020 (Fig 1). IRs of all three outcomes returned to pre-pandemic levels in summer 2020. However, IRs of asthma decreased by 5% (IRR: 0.95, 95% CI: 0.94, 0.97) in fall 2020 and by 10% (IRR: 0.90, 95% CI: 0.89, 0.92) in winter 2021. In addition, there was an increase in the IRs of all three outcomes in the post-pandemic periods compared to the corresponding timeframe in 2018: IRs of asthma increased by up to 12% since spring 2021; IRs of AD increased by up to 35% since summer 2020; IRs of MS increased by up to 24% since spring 2021 (Fig 1 and S1 Fig).

**Fig 2:**
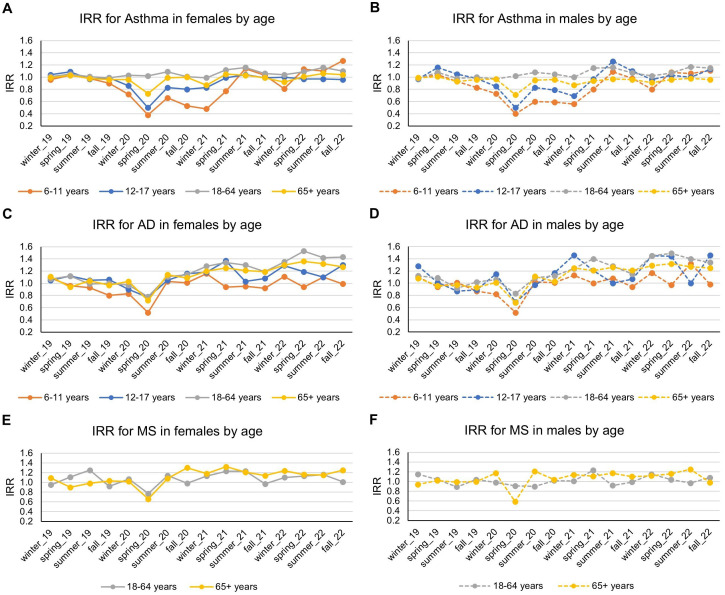
Incidence rate ratios (IRR) of asthma, atopic dermatitis (AD), and multiple sclerosis (MS) in seasonal cohorts from 2019 to 2022 compared to the corresponding timeframe in 2018 by age and sex in Optum^®^ CDM. IRRs were calculated to compare the IRs in age and sex subgroups in seasonal cohorts from 2019 to 2022 to the IRs in the corresponding age and sex subgroups in the corresponding timeframe in 2018 (reference).

S2 Fig shows the IRs for asthma, AD, and MS in each seasonal cohort by age and sex and Fig 2 presents the IRRs comparing the IRs in 2019–2022 to those in 2018. For asthma, the reduction in IRs in spring 2020 was observed for all age and sex subgroups, except for adults aged 18–64 years (Fig 2). For those between 6–11 years, IRs of asthma were 62% (IRR: 0.38, 95% CI: 0.32, 0.44) less for females and 60% (IRR: 0.40, 95% CI: 0.36, 0.45) less for males in spring 2020 relative to females and males in spring 2018, respectively. IRs of asthma decreased by 50% for both females (IRR: 0.50, 95% CI: 0.44, 0.56) and males (IRR: 0.50, 95% CI: 0.45, 0.57) in adolescents aged 12–17 years, and decreased by 27% to 29% in senior adults aged ≥65 years (IRR: 0.73, 95% CI: 0.70, 0.75 in females and IRR: 0.71, 95% CI: 0.68, 0.74 in males) (S2 Fig). For AD, IRs declined during spring 2020 in all age and sex subgroups while young children aged 6–11 years were more affected (IRR: 0.52 in both females and in males) compared to other subgroups (IRRs ranged 0.68–0.83). The reduction in IRs in MS was more affected in older adults aged ≥65 years (IRR: 0.66 in females and 0.59 in males) than younger adults aged 18–64 years (IRR: 0.77 in females and 0.91 in males). IRs of MS in those under 18 years were not calculated due to small counts. For all three outcomes, there was no sex difference in the changes of IRs in different periods relative to those in spring 2018 (Fig 2 and S2 Fig).

## Discussion

In this study, we examined the impact of the COVID-19 pandemic on newly diagnosed cases of asthma, AD, and MS observed in an administrative health claims database in the US. We found that compared to the pre-pandemic levels, there was a significant reduction in the observed IRs in spring (March to May) 2020, when stay-at-home orders and hospital restrictions were implemented [3,20]. The decline in estimated incidence during the immediate pandemic period was more pronounced in children, adolescents, and senior adults than younger adults. Since summer 2020, a return to baseline or even greater IRs for all three outcomes was observed.

Although previous studies observed reduced healthcare utilization in asthma [21–25], AD [26,27], and MS patients [28,29] during the pandemic, our study extends this evidence by demonstrating how this reduction translated into a notable decline in incidence estimates from March to May 2020. Substantial reductions in asthma-related emergency department (ED) visits, hospitalizations, and outpatient encounters during the first few months of COVID-19 were widely reported across multiple healthcare settings [21–25], along with decreases in dermatological hospitalizations and ED consultations in Italy and Poland [26,27], and a 16.8% reduction in hospital admissions for incident MS cases in Germany [28]. These declines likely reflect delays in diagnosis due to limited access to healthcare services from stay-at-home notices [2,3,30], restrictions on non-urgent care [31], and redeployment of healthcare workforce to assist with the COVID-19 emergency [32]. From the patient perspective, fear of becoming infected with COVID-19 during in-person medical visits may have led to canceled or deferred medical care [4,33].

In the present study, IRs rebounded to pre-pandemic levels after May 2020, although the timing of the “catch-up” differed by outcomes. For AD and MS, IRs returned to baseline levels in summer 2020, which was aligned with previous studies [6,34]. In contrast, IRs of asthma dropped by 5%−10% from September 2020 through February 2021, and did not return to pre-pandemic levels until spring 2021, which seemed mainly driven by the slower recovery of IRs in children and adolescents (**Fig 2**). Factors other than healthcare avoidance might have contributed to the observed decline, particularly in pediatric populations [35]. Mitigation efforts (e.g., schools and childcare facility closures) and behavior changes (e.g., face mask wearing) likely reduced transmission of respiratory pathogens, such as rhinovirus [24,35], a trigger for asthma onset and exacerbations [36]. Therefore, our results suggested an artificial decline during the early phase of the pandemic, which may pose challenges in the estimation and interpretation of long-term incidence trends. During the study planning stage, researchers may consider excluding the 2020 data from trend analysis [37,38]. Our previous work suggested that sufficient post-pandemic data (2021 and onward) should be accrued to mitigate the impact of the COVID-19 pandemic on cumulative incidence estimation [39].

Age- and sex-stratified analyses showed that children, adolescents, and senior adults above 65 years experienced larger reductions in IRs compared to adults aged 18−64 years, and this was consistent with other studies [7,40]. A US claims-based study similarly found that there were greater decreases in non-COVID-19 related inpatient, ED, or outpatient visits among children and seniors than middle-aged adults (45−64 years) [7]. These age differences could be attributed to the parental avoidance of or hesitancy to access healthcare services for their children [41,42], as well as medical care avoidance in older adults who were more likely to have pre-existing conditions (e.g., diabetes [43]) considered risk factors for poor COVID-19 outcomes [44]. While previous studies reported that females were likely to delay medical care during the pandemic [45,46], they primarily focused on general healthcare utilization without differentiating disease-specific medical services. We did not find sex differences in the changes of IRs for the three outcomes, and this finding was consistent with previous findings for asthma and MS [24,28]. In a study using electronic health records in Philadelphia, the total number of pediatric asthma encounters decreased in March-May 2020, and the proportion of encounters by females did not change compared to pre-pandemic levels [24]. Sex distribution of hospitalized patients with incident MS was found similar before and during the pandemic in a German nationwide study [28]. The sex difference in healthcare utilization change for incident AD cases during the pandemic remains understudied. More research is needed to replicate our findings and further explore sex disparities in disease incidence in the context of the pandemic.

There was an increase in IRs, especially for AD, in 2021−2022 compared to 2018. The “catch-up” of delayed access to healthcare due to the pandemic may partially explain this temporal increase. Furthermore, there seemed to be an increase in the incidence of AD over time, even before the COVID-19 pandemic [47,48]. A true post-pandemic increase in IRs may also be plausible. Specifically, there has been evidence suggesting the increased risk of allergic diseases in patients with previous COVID-19 infection [49,50]. Repeated hand washing and sanitizing may lead to disruption of the skin barrier, increased skin irritation and subsequent hand eczema [51]. Besides, adverse psychological consequences during the COVID-19 [52,53] may have an impact on the risk of common stress‐responsive skin conditions, such as AD [54,55]. For MS, COVID-19 infection may be associated with inflammation-induced reactivation of the Epstein–Barr virus [56,57], which is believed to be a trigger for the onset of MS [58]. The long-term temporal incidence trends of these diseases could be explored in further research.

Our study had several limitations. First, misclassification of the outcomes cannot be ruled out. The use of two diagnostic codes for asthma and AD identification may have missed milder incident cases. MS cases without a dispensing for DMT may have been misclassified as non-cases despite a validated algorithm. Furthermore, the use of a one-year “wash-out” period may result in misclassification of prevalent cases as incident ones [59]. Second, lack of direct measures of healthcare restrictions or individual-level healthcare-seeking behavior limit our ability to establish causal attribution between these factors and the results observed. However, this study was not aimed at investigating the causal effect of specific restrictions. The concurrent declines in incidence observed in spring 2020 coincided with widespread stay-at-home orders and healthcare service restrictions, and this likely represented underascertainment due to reduced healthcare encounters. Third, although we performed stratified analyses by age and sex, we did not account for other important confounders, such as socioeconomic status, region of residence, and comorbidities, some of which are unavailable in claims database and may have been associated with healthcare utilization [60,61], health-seeking behaviors [46,62], and risk of non-COVID-19-related diseases [63,64]. Finally, although our database included claims from a large study population, it was primarily employer-based and may not be representative of the overall population. Our findings may not be generalized to other populations, such as individuals with Medicare or Medicaid and those lacking insurance.

## Conclusions

Our findings demonstrate that the COVID-19 pandemic resulted in an apparent reduction in observed incident case identification during spring 2020, likely due to delayed access to healthcare resulting from restricted medical encounters. Researchers should exercise caution when planning claims-based studies, particularly regarding the timeframes of data used. Beyond healthcare utilization changes, COVID-19 infection itself and other pandemic-related changes may also influence long-term disease incidence trends, warranting further research.

## Supporting information

S1 Table List of codes for disease modifying therapies for multiple sclerosis. (DOCX)

S2 Table Population characteristics of seasonal cohorts in 2018. (DOCX)

S1 Fig Incidence rates (IR) of asthma, atopic dermatitis (AD), and multiple sclerosis (MS) in seasonal cohorts from 2018 to 2022 in Optum^®^ CDM. Standardized IRs were calculated using a direct method with the population composition by age and sex in the United States in 2022 as the reference population.
(DOCX)

S2 Fig Incidence rates (IR) and incidence rate ratios (IRR) of asthma, atopic dermatitis (AD), and multiple sclerosis (MS) in seasonal cohorts from 2018 to 2022 by age and sex in Optum^®^ CDM. Panels A-B, D-E, and G-H: IRs for asthma, AD, and MS were calculated by age and sex from 2018 to 2022. Panels C, F, and I: IRRs were calculated to compare the IRs in age and sex subgroups in spring 2020 to the IRs in the corresponding age and sex subgroups in spring 2018. Dots represent the IRRs and error bars represent the 95% confidence intervals. Dash lines represent an IRR of 1.
(DOCX)

## References

[1] Hartnett KP, Kite-Powell A, DeVies J, Coletta MA, Boehmer TK, Adjemian J, et al. Impact of the COVID-19 Pandemic on Emergency Department Visits - United States, January 1, 2019-May 30, 2020. MMWR Morb Mortal Wkly Rep. 2020;69(23):699–704. 32525856 10.15585/mmwr.mm6923e1 PMC7315789

[2] Whaley CM, Pera MF, Cantor J, Chang J, Velasco J, Hagg HK, et al. Changes in health services use among commercially insured US populations during the COVID-19 pandemic. JAMA Network Open. 2020;3(11):e2024984-e. doi: 10.1001/jamanetworkopen.2020.24984 PMC7645698 33151319

[3] Ziedan E, Simon KI, Wing C. Effects of state COVID-19 closure policy on non-COVID-19 health care utilization. National Bureau of Economic Research. 2020. https://www.nber.org/papers/w27621

[4] Czeisler MÉ. Delay or avoidance of medical care because of COVID-19–related concerns—United States, June 2020. MMWR Morbidity and Mortality Weekly Report. 2020;69. 10.15585/mmwr.mm6936a4 PMC7499838 32915166

[5] Baum A, Schwartz MD. Admissions to Veterans Affairs Hospitals for Emergency Conditions During the COVID-19 Pandemic. JAMA. 2020;324(1):96–9. doi: 10.1001/jama.2020.9972 32501493 PMC7275263

[6] Birkmeyer JD, Barnato A, Birkmeyer N, Bessler R, Skinner J. The impact of the COVID-19 pandemic on hospital admissions in the United States. Health Aff (Millwood). 2020;39(11):2010–7. 32970495 10.1377/hlthaff.2020.00980 PMC7769002

[7] Kim Y, Gordon A, Rowerdink K, Herrera Scott L, Chi W. The Impact of the COVID-19 Pandemic on Health Care Utilization Among Insured Individuals With Common Chronic Conditions. Med Care. 2022;60(9):673–9. doi: 10.1097/MLR.0000000000001747 35866561 PMC9365072

[8] Dondi A, Betti L, Carbone C, Dormi A, Paglione M, Rinaldi M, et al. Understanding the environmental factors related to the decrease in Pediatric Emergency Department referrals for acute asthma during the SARS-CoV-2 pandemic. Pediatr Pulmonol. 2022;57(1):66–74. doi: 10.1002/ppul.25695 34606693 PMC8661783

[9] Xu S, Glenn S, Sy L, Qian L, Hong V, Ryan DS, et al. Impact of the COVID-19 Pandemic on Health Care Utilization in a Large Integrated Health Care System: Retrospective Cohort Study. J Med Internet Res. 2021;23(4):e26558. doi: 10.2196/26558 33882020 PMC8086778

[10] Pifarré I Arolas H, Vidal-Alaball J, Gil J, López F, Nicodemo C, Saez M. Missing Diagnoses during the COVID-19 Pandemic: A Year in Review. Int J Environ Res Public Health. 2021;18(10):5335. doi: 10.3390/ijerph18105335 34067807 PMC8156815

[11] Williams R, Jenkins DA, Ashcroft DM, Brown B, Campbell S, Carr MJ, et al. Diagnosis of physical and mental health conditions in primary care during the COVID-19 pandemic: a retrospective cohort study. Lancet Public Health. 2020;5(10):e543–50. doi: 10.1016/S2468-2667(20)30201-2 32979305 PMC7511209

[12] Howlader N, Bhattacharya M, Scoppa S, Miller D, Noone A-M, Negoita S, et al. Cancer and COVID-19: US cancer incidence rates during the first year of the pandemic. J Natl Cancer Inst. 2024;116(2):208–15. doi: 10.1093/jnci/djad205 37796818 PMC10852612

[13] Kempers EK, Chen Q, Visser C, van Gorp ECM, Klok FA, Cannegieter SC, et al. Changes in incidence of hospitalization for cardiovascular diseases during the COVID-19 pandemic in The Netherlands in 2020. Sci Rep. 2023;13(1):12832. doi: 10.1038/s41598-023-39573-w 37553430 PMC10409797

[14] Hamann CR, Andersen YMF, Engebretsen KA, Skov L, Silverberg JI, Egeberg A, et al. The effects of season and weather on healthcare utilization among patients with atopic dermatitis. J Eur Acad Dermatol Venereol. 2018;32(10):1745–53. doi: 10.1111/jdv.15023 29706020

[15] Chen C-H, Xirasagar S, Lin H-C. Seasonality in adult asthma admissions, air pollutant levels, and climate: a population-based study. J Asthma. 2006;43(4):287–92. doi: 10.1080/02770900600622935 16809242

[16] Handel AE, Disanto G, Jarvis L, McLaughlin R, Fries A, Ebers GC, et al. Seasonality of admissions with multiple sclerosis in Scotland. Eur J Neurol. 2011;18(8):1109–11. doi: 10.1111/j.1468-1331.2010.03318.x 21749578

[17] Culpepper WJ, Marrie RA, Langer-Gould A, Wallin MT, Campbell JD, Nelson LM, et al. Validation of an algorithm for identifying MS cases in administrative health claims datasets. Neurology. 2019;92(10):e1016–28. doi: 10.1212/WNL.0000000000007043 30770432 PMC6442008

[18] Brown LD, Cai TT, DasGupta A. Interval Estimation for a Binomial Proportion. Statist Sci. 2001;16(2). doi: 10.1214/ss/1009213286

[19] U.S. Census Bureau. Single-race Population Estimates, United States 2022. https://wonder.cdc.gov/wonder/help/single-race.html

[20] Wu J, Smith S, Khurana M, Siemaszko C, DeJesus-Banos B. Stay-at-home orders across the country 2020. https://www.nbcnews.com/health/health-news/here-are-stay-home-orders-across-country-n1168736 2020.

[21] Kenyon CC, Hill DA, Henrickson SE, Bryant-Stephens TC, Zorc JJ. Initial effects of the COVID-19 pandemic on pediatric asthma emergency department utilization. J Allergy Clin Immunol Pract. 2020;8(8):2774–2776.e1. doi: 10.1016/j.jaip.2020.05.045 32522565 PMC7483361

[22] Davies GA, Alsallakh MA, Sivakumaran S, Vasileiou E, Lyons RA, Robertson C, et al. Impact of COVID-19 lockdown on emergency asthma admissions and deaths: national interrupted time series analyses for Scotland and Wales. Thorax. 2021;76(9):867–73. doi: 10.1136/thoraxjnl-2020-216380 33782079

[23] Gaffney A, Himmelstein DU, Woolhandler S. Population-level trends in asthma and chronic obstructive pulmonary disease emergency department visits and hospitalizations before and during the coronavirus disease 2019 pandemic in the United States. Ann Allergy Asthma Immunol. 2023;131(6):737–744.e8. doi: 10.1016/j.anai.2023.08.016 37619778

[24] Taquechel K, Diwadkar AR, Sayed S, Dudley JW, Grundmeier RW, Kenyon CC. Pediatric Asthma Health Care Utilization, Viral Testing, and Air Pollution Changes During the COVID-19 Pandemic. The Journal of Allergy and Clinical Immunology: In Practice. 2020;8(10):3378–87.e11. 32827728 10.1016/j.jaip.2020.07.057 PMC7438361

[25] Henry SS, Duong KE, Cabana MD, Duong TQ. Effects of COVID-19 pandemic on incidence of asthma exacerbation in an urban population. Sci Rep. 2026;16(1):10352. doi: 10.1038/s41598-026-41311-x 41735473 PMC13031412

[26] Białynicki-Birula R, Siemasz I, Otlewska A, Matusiak Ł, Szepietowski JC. Influence of COVID-19 pandemic on hospitalizations at the tertiary dermatology department in south-west Poland. Dermatol Ther. 2020;33(4):e13738. doi: 10.1111/dth.13738 32478949 PMC7300546

[27] Isoletta E, Vassallo C, Brazzelli V, Giorgini C, Tomasini CF, Sabena A, et al. Emergency accesses in Dermatology Department during the Covid-19 pandemic in a referral third level center in the north of Italy. Dermatol Ther. 2020;33(6):e14027. doi: 10.1111/dth.14027 32681752 PMC7404501

[28] Richter D, Faissner S, Bartig D, Tönges L, Hellwig K, Ayzenberg I, et al. The impact of the COVID-19 pandemic on hospitalizations and plasmapheresis therapy in multiple sclerosis and neuromyelitis optica spectrum disorder: a nationwide analysis from Germany. Ther Adv Neurol Disord. 2021;14. doi: 10.1177/17562864211030656 34285719 PMC8267031

[29] Prosperini L, Arrambide G, Celius EG, Goletti D, Killestein J, Kos D, et al. COVID-19 and multiple sclerosis: challenges and lessons for patient care. Lancet Reg Health Eur. 2024;44:100979. doi: 10.1016/j.lanepe.2024.100979 39429966 PMC11486927

[30] Giannouchos TV, Biskupiak J, Moss MJ, Brixner D, Andreyeva E, Ukert B. Trends in outpatient emergency department visits during the COVID-19 pandemic at a large, urban, academic hospital system. Am J Emerg Med. 2021;40:20–6. doi: 10.1016/j.ajem.2020.12.009 33338676 PMC7725055

[31] Centers for Medicare & Medicaid Services. Non-Emergent, Elective Medical Services, and Treatment Recommendations. 2020. https://www.cms.gov/files/document/cms-non-emergent-elective-medical-recommendations.pdf

[32] Vera San Juan N, Clark SE, Camilleri M, Jeans JP, Monkhouse A, Chisnall G, et al. Training and redeployment of healthcare workers to intensive care units (ICUs) during the COVID-19 pandemic: a systematic review. BMJ Open. 2022;12(1):e050038. doi: 10.1136/bmjopen-2021-050038 34996785 PMC8753114

[33] Splinter MJ, Velek P, Ikram MK, Kieboom BCT, Peeters RP, Bindels PJE, et al. Prevalence and determinants of healthcare avoidance during the COVID-19 pandemic: A population-based cross-sectional study. PLoS Med. 2021;18(11):e1003854. doi: 10.1371/journal.pmed.1003854 34813591 PMC8610236

[34] Engelbrecht K, Roy S, Capkun G, Kahler K, Olson M. Impact of the COVID-19 pandemic on healthcare resource utilization across selected disease areas in the USA. J Comp Eff Res. 2022;11(11):815–28. doi: 10.2217/cer-2022-0059 35699096 PMC9251631

[35] Ye D, Gates A, Radhakrishnan L, Mirabelli MC, Flanders WD, Sircar K. Changes in asthma emergency department visits in the United States during the COVID-19 pandemic. J Asthma. 2023;60(8):1601–7. doi: 10.1080/02770903.2023.2165445 36608267 PMC10293019

[36] Jackson DJ, Gern JE. Rhinovirus Infections and Their Roles in Asthma: Etiology and Exacerbations. J Allergy Clin Immunol Pract. 2022;10(3):673–81. doi: 10.1016/j.jaip.2022.01.006 35074599 PMC10314805

[37] Mariotto AB, Feuer EJ, Howlader N, Chen H-S, Negoita S, Cronin KA. Interpreting cancer incidence trends: challenges due to the COVID-19 pandemic. J Natl Cancer Inst. 2023;115(9):1109–11. doi: 10.1093/jnci/djad086 37220901 PMC10483261

[38] Surveillance E. Impact of COVID on the April 2024 SEER Data Release. 2024. https://seer.cancer.gov/data/covid-impact.html

[39] Barberio J, Zhu K, Harikrishnan V, Li X, Sinnott S-J. Assessing the Impact of the COVID-19 Pandemic on Relative Incidence Rates in United States Claims Data. Pharmacoepidemiol Drug Saf. 2025;34(11):e70246. doi: 10.1002/pds.70246 41139508

[40] Johnson KJ, Goss CW, Thompson JJ, Trolard AM, Maricque BB, Anwuri V, et al. Assessment of the impact of the COVID-19 pandemic on health services use. Public Health Pract (Oxf). 2022;3:100254. doi: 10.1016/j.puhip.2022.100254 35403073 PMC8979834

[41] Nicholson E, McDonnell T, Conlon C, Barrett M, Cummins F, Hensey C, et al. Parental Hesitancy and Concerns around Accessing Paediatric Unscheduled Healthcare during COVID-19: A Cross-Sectional Survey. Int J Environ Res Public Health. 2020;17(24):9264. doi: 10.3390/ijerph17249264 33322332 PMC7763208

[42] Lazzerini M, Barbi E, Apicella A, Marchetti F, Cardinale F, Trobia G. Delayed access or provision of care in Italy resulting from fear of COVID-19. Lancet Child Adolesc Health. 2020;4(5):e10–1. doi: 10.1016/S2352-4642(20)30108-5 32278365 PMC7146704

[43] Centers for Disease Control and Prevention. National diabetes statistics report: estimates of diabetes and its burden in the United States. https://stacks.cdc.gov/view/cdc/148231 2023.

[44] Apicella M, Campopiano MC, Mantuano M, Mazoni L, Coppelli A, Del Prato S. COVID-19 in people with diabetes: understanding the reasons for worse outcomes. Lancet Diabetes Endocrinol. 2020;8(9):782–92. doi: 10.1016/S2213-8587(20)30238-2 32687793 PMC7367664

[45] Zhong S, Huisingh-Scheetz M, Huang ES. Delayed medical care and its perceived health impact among US older adults during the COVID-19 pandemic. J Am Geriatr Soc. 2022;70(6):1620–8. doi: 10.1111/jgs.17805 35393637 PMC9177755

[46] Jones AN, Power MC. Pre-pandemic factors associated with delayed health care among US older adults during the COVID-19 pandemic. J Med Access. 2023;7. doi: 10.1177/27550834231202860 37872971 PMC10590541

[47] Deckers IAG, McLean S, Linssen S, Mommers M, van Schayck CP, Sheikh A. Investigating international time trends in the incidence and prevalence of atopic eczema 1990-2010: a systematic review of epidemiological studies. PLoS One. 2012;7(7):e39803. doi: 10.1371/journal.pone.0039803 22808063 PMC3394782

[48] Henriksen L, Simonsen J, Haerskjold A, Linder M, Kieler H, Thomsen SF, et al. Incidence rates of atopic dermatitis, asthma, and allergic rhinoconjunctivitis in Danish and Swedish children. J Allergy Clin Immunol. 2015;136(2):360–6.e2. doi: 10.1016/j.jaci.2015.02.003 25828267

[49] Oh J, Lee M, Kim M, Kim HJ, Lee SW, Rhee SY, et al. Incident allergic diseases in post-COVID-19 condition: multinational cohort studies from South Korea, Japan and the UK. Nat Commun. 2024;15(1):2830. doi: 10.1038/s41467-024-47176-w 38565542 PMC10987608

[50] Schmitt J, Ehm F, Vivirito A, Wende D, Batram M, Loser F, et al. Large cohort study shows increased risk of developing atopic dermatitis after COVID-19 disease. Allergy. 2024;79(1):232–4. doi: 10.1111/all.15827 37469301

[51] Blicharz L, Czuwara J, Samochocki Z, Goldust M, Chrostowska S, Olszewska M, et al. Hand eczema-A growing dermatological concern during the COVID-19 pandemic and possible treatments. Dermatol Ther. 2020;33(5):e13545. doi: 10.1111/dth.13545 32384196 PMC7261986

[52] Vahratian A. Symptoms of anxiety or depressive disorder and use of mental health care among adults during the COVID-19 pandemic—United States, August 2020–February 2021. MMWR Morbidity and Mortality Weekly Report. 2021;70. 10.15585/mmwr.mm7013e2 PMC8022876 33793459

[53] Park CL, Russell BS, Fendrich M, Finkelstein-Fox L, Hutchison M, Becker J. Americans’ COVID-19 Stress, Coping, and Adherence to CDC Guidelines. J Gen Intern Med. 2020;35(8):2296–303. doi: 10.1007/s11606-020-05898-9 32472486 PMC7259430

[54] Garcovich S, Bersani FS, Chiricozzi A, De Simone C. Mass quarantine measures in the time of COVID-19 pandemic: psychosocial implications for chronic skin conditions and a call for qualitative studies. J Eur Acad Dermatol Venereol. 2020;34(7):e293–4. doi: 10.1111/jdv.16535 32330329 PMC7267356

[55] Tefft KR, Balboul S, Safai B, Cline A, Marmon S. Diagnosis of stress-associated dermatologic conditions in New York City safety-net hospitals during the COVID-19 pandemic. J Am Acad Dermatol. 2022;87(5):e177–9. doi: 10.1016/j.jaad.2022.05.066 35780941 PMC9482688

[56] Su Y, Yuan D, Chen DG, Ng RH, Wang K, Choi J, et al. Multiple early factors anticipate post-acute COVID-19 sequelae. Cell. 2022;185(5):881–895.e20. doi: 10.1016/j.cell.2022.01.014 35216672 PMC8786632

[57] Gold JE, Okyay RA, Licht WE, Hurley DJ. Investigation of Long COVID Prevalence and Its Relationship to Epstein-Barr Virus Reactivation. Pathogens. 2021;10(6):763. doi: 10.3390/pathogens10060763 34204243 PMC8233978

[58] Aloisi F, Giovannoni G, Salvetti M. Epstein-Barr virus as a cause of multiple sclerosis: opportunities for prevention and therapy. Lancet Neurol. 2023;22(4):338–49. doi: 10.1016/S1474-4422(22)00471-9 36764322

[59] Rassen JA, Bartels DB, Schneeweiss S, Patrick AR, Murk W. Measuring prevalence and incidence of chronic conditions in claims and electronic health record databases. Clin Epidemiol. 2018;11:1–15. doi: 10.2147/CLEP.S181242 30588119 PMC6301730

[60] Lowe J, Brown I, Duriseti R, Gallegos M, Ribeira R, Pirrotta E, et al. Emergency department access during COVID-19: disparities in utilization by race/ethnicity, insurance, and income. West J Emerg Med. 2021;22(3):552–60. 34125026 10.5811/westjem.2021.1.49279 PMC8203020

[61] Amezcua L, Rivera VM, Vazquez TC, Baezconde-Garbanati L, Langer-Gould A. Health Disparities, Inequities, and Social Determinants of Health in Multiple Sclerosis and Related Disorders in the US. JAMA Neurol. 2021;78(12):1515. doi: 10.1001/jamaneurol.2021.3416 34605866

[62] Håkansson KEJ, Backer V, Ulrik CS. Socioeconomic status is associated with healthcare seeking behaviour and disease burden in young adults with asthma - A nationwide cohort study. Chron Respir Dis. 2022;19. doi: 10.1177/14799731221117297 35938497 PMC9364195

[63] Mielck A, Reitmeir P, Wjst M. Severity of childhood asthma by socioeconomic status. Int J Epidemiol. 1996;25(2):388–93. doi: 10.1093/ije/25.2.388 9119565

[64] Boorgu DSSK, Venkatesh S, Lakhani CM, Walker E, Aguerre IM, Riley C, et al. The impact of socioeconomic status on subsequent neurological outcomes in multiple sclerosis. Mult Scler Relat Disord. 2022;65:103994. doi: 10.1016/j.msard.2022.103994 35780727 PMC9444968

