# Supplementary material for: Trends in the incidence of asthma, atopic dermatitis, and multiple sclerosis before, during, and after the COVID-19 pandemic in a US claims database
Source: PLoS One. 2026 Jul 30;21(7):e0355103. doi: 10.1371/journal.pone.0355103 (PMC13422859; doi:10.1371/journal.pone.0355103)
Supplement: S1 Table — (DOCX) [file pone.0355103.s001.docx]

# S1 Table. List of codes for disease modifying therapies for multiple sclerosis

| **Generic Name** | **Brand Name** | **Drug Class** | **HCPCS code** | **NDC** |
| --- | --- | --- | --- | --- |
| Alemtuzumab | LEMTRADA | High Efficacy | J0202 | 58468-0200 |
| Daclizumab | ZINBRYTA | High Efficacy | J7513 | 0074-0033  0074-0034 |
| Natalizumab | TYSABRI | High Efficacy | J2323 | 64406-008 |
| Ocrelizumab | OCREVUS | High Efficacy | J2350, C9494 | 50242-150 |
| Rituximab | Rituxan, ® Riabni,® Truxima® | High Efficacy | J9310, C9467, J9311, J9312 | 50242-051,  50242-053,  50242-108,  50242-109,  55513-224,  55513-326,  63459-103,  63459-104 |
| Mitoxantrone | Novantrone ® | High Efficacy | J9293 | 61703-343  63323-132 |
| Fingolimod | Gilenya ®, Tascenso Odt ® | High Efficacy | NA | 0078-0607,  0078-0965,  0378-4525,  16729-342,  31722-889,  43547-003,  43598-285,  60505-4332,  62756-064,  64980-449.  67877-476,  68382-912,  68462-166,  70709-062,  70709-065,  70771-1603,  73086-300 |
| Ofatumumab | Kesimpta ® | High Efficacy | J9302 | 0078-0669,  0078-0690,  0078-1007 |
| Ponesimod | PONVORY ® | High Efficacy | NA | 50458-707,   50458-720 |
| Siponimod | Mayzent ® | High Efficacy | NA | 0078-0979,  0078-0986,  0078-1014 |
| Ozanimod | Zeposia ® | High Efficacy | NA | 59572-810,  59572-820,  59572-890 |
| Cladribine | MAVENCLAD | High Efficacy | J9065 | 0143-9871,   42658-010,   44087-4000,  63323-140 |
| Dimethyl Fumarate | TECFIDERA | Oral | NA | 0093-9219,  0378-0396,  0378-0399,  16729-416,  16729-417,  16729-418,  24979-127,  24979-128,  31722-657,  31722-658,  31722-680,  43547-024,  43547-025,  43598-429,  43598-430,  50090-5288,  50090-6722,  51407-441,  51407-442,  59651-083,  59651-084,  64406-005,  64406-006,  64406-007,  67877-555,  67877-556,  67877-557,  68462-307,  68462-308,  68462-570,  69097-322,  69097-323,  69097-552,  69238-1318,  69238-1319,  69238-1626,  69539-042,  69539-043,  69539-240,  70512-852,  70512-853,  70710-1204,  70710-1205,  70710-1416,  70771-1530,  70771-1531 |
| Teriflunomide | AUBAGIO | Oral | NA | 0378-0627,  0378-0628,  0480-3156,  0480-3157,  0781-5747,  0781-5755,  16729-399,  16729-400,  31722-246,  31722-247,  42291-830,  42291-831,  43598-281,  43598-282,  46708-313,  46708-314,  51991-881,  51991-882,  58468-0210,  58468-0211,  59651-054,  59651-055,  60505-4477,  60505-4478,  62332-313,  62332-314,  68462-423,  68462-424,  69025-129,  69025-130,  69238-1303,  69238-1304,  69339-169,  69339-170,  69539-032,  69539-033,  69539-315,  69539-316,  70377-017,  70377-018,  70512-850,  70512-851,  70710-1114,  70710-1115,  70771-1010,  70771-1011 |
| Glatiramer Acetate | COPAXONE, GLATOPA | Platform Injectables | J1595 | 0378-6960,  0378-6961,  0781-3234,  0781-3250,  63629-8815,  63629-8816,  68546-317,  68546-325 |
| Interferon β-1A | AVONEX, REBIF | Platform Injectables | J1826 | 44087-0022,  44087-0044,  44087-0188,  44087-3322,  44087-3344,  44087-8822,  59627-002,  59627-003,  59627-222,  59627-333 |
| Interferon β-1B | BETASERON, EXTAVIA | Platform Injectables | J1830 | 0078-0569,  50419-524 |
| Peginterferon β-1A | PLEGRIDY | Platform Injectables | NA | 64406-011,  64406-012,  64406-015,  64406-016,  64406-017 |
