# Supplementary material for: Trends in the incidence of asthma, atopic dermatitis, and multiple sclerosis before, during, and after the COVID-19 pandemic in a US claims database
Source: PLoS One. 2026 Jul 30;21(7):e0355103. doi: 10.1371/journal.pone.0355103 (PMC13422859; doi:10.1371/journal.pone.0355103)
Supplement: S2 Table — (DOCX) [file pone.0355103.s002.docx]

**S2 Table. Population characteristics of seasonal cohorts in 2018**

| **Characteristics** | **Seasonal cohorts (denominators) for the asthma outcome** | | | |
| --- | --- | --- | --- | --- |
|  | **Winter 2018** | **Spring 2018** | **Summer 2018** | **Fall 2018** |
| Total N | 10,349,868 | 10,986,712 | 10,998,434 | 11,027,431 |
| Age, mean (SD) | 49.37 (22.63) | 51.10 (22.96) | 50.96 (22.91) | 50.93 (22.86) |
| Age groups, n (%) |  |  |  |  |
| 6-11 years | 537,351 (5.19) | 527,889 (4.80) | 531,595 (4.83) | 534,149 (4.84) |
| 12-17 years | 594,470 (5.74) | 588,327 (5.35) | 591,023 (5.37) | 592,938 (5.38) |
| 18-64years | 5,894,605 (56.95) | 5,943,362 (54.10) | 5,968,124 (54.26) | 5,975,567 (54.19) |
| ≥65 years | 3,323,442 (32.11) | 3,927,134 (35.74) | 3,907,692 (35.53) | 3,924,777 (35.59) |
| Sex, n (%) |  |  |  |  |
| Female | 5,275,078 (50.97) | 5,623,021 (51.18) | 5,630,652 (51.20) | 5,647,483 (51.21) |
| Male | 5,074,790 (49.03) | 5,363,691 (48.82) | 5,367,782 (48.80) | 5,379,948 (48.79) |
| Race and ethnicity, n (%) |  |  |  |  |
| Asian | 573,258 (5.54) | 598,709 (5.45) | 602,793 (5.48) | 604,012 (5.48) |
| Black | 1,008,428 (9.74) | 1,077,606 (9.81) | 1,079,563 (9.82) | 1,075,092 (9.75) |
| Hispanic | 1,276,420 (12.33) | 1,349,792 (12.29) | 1,356,263 (12.33) | 1,357,953 (12.31) |
| Other | 401,463 (3.88) | 431,669 (3.93) | 434,735 (3.95) | 446,930 (4.05) |
| White | 7,090,299 (68.51) | 7,528,936 (68.53) | 7,525,080 (68.42) | 7,543,444 (68.41) |
| **Characteristics** | **Seasonal cohorts (denominators) for the atopic dermatitis (AD) outcome** | | | |
|  | **Winter 2018** | **Spring 2018** | **Summer 2018** | **Fall 2018** |
| Total N | 10,862,371 | 11,524,682 | 11,526,367 | 11,553,621 |
| Age, mean (SD) | 49.52 (22.62) | 51.25 (22.95) | 51.11 (22.89) | 51.07 (22.84) |
| Age groups, n (%) |  |  |  |  |
| 6-11 years | 563412 (5.19) | 551184 (4.78) | 554637 (4.81) | 557,103 (4.82) |
| 12-17 years | 626339 (5.77) | 619445 (5.37) | 621317 (5.39) | 623,015 (5.39) |
| 18-64years | 6155751 (56.67) | 6200037 (53.8) | 6222911 (53.99) | 6,229,859 (53.92) |
| ≥65 years | 3516869 (32.38) | 4154016 (36.04) | 4127502 (35.81) | 4,143,644 (35.86) |
| Sex, n (%) |  |  |  |  |
| Female | 5,603,351 (51.58) | 5,969,294 (51.80) | 5,971,818 (51.81) | 5,988,093 (51.83) |
| Male | 5,259,020 (48.42) | 5,555,388 (48.20) | 5,554,549 (48.19) | 5,565,528 (48.17) |
| Race and ethnicity, n (%) |  |  |  |  |
| Asian | 587,725 (5.41) | 613,661 (5.32) | 617,393 (5.36) | 618,226 (5.35) |
| Black | 1,075,319 (9.9) | 1,147,182 (9.95) | 1,148,633 (9.97) | 1,143,990 (9.9) |
| Hispanic | 1,330,808 (12.25) | 1,407,924 (12.22) | 1414069 (12.27) | 1,415,269 (12.25) |
| Other | 420,018 (3.87) | 451,649 (3.92) | 454,185 (3.94) | 466,260 (4.04) |
| White | 7,448,501 (68.57) | 7,904,266 (68.59) | 7,892,087 (68.47) | 7,909,876 (68.46) |
| **Characteristics** | **Seasonal cohorts (denominators) for the multiple sclerosis (MS) outcome** | | | |
|  | **Winter 2018** | **Spring 2018** | **Summer 2018** | **Fall 2018** |
| Total N | 10,927,353 | 11,602,992 | 11,610,954 | 11,640,723 |
| Age, mean (SD) | 49.45 (22.67) | 51.18 (23) | 51.04 (22.94) | 51.00 (22.90) |
| Age groups, n (%) |  |  |  |  |
| 6-11 years | 577411 (5.28) | 566279 (4.88) | 569827 (4.91) | 572,305 (4.92) |
| 12-17 years | 636,105 (5.82) | 630383 (5.43) | 632540 (5.45) | 634,381 (5.45) |
| 18-64years | 6,178,747 (56.54) | 6227324 (53.67) | 6253545 (53.86) | 6,261,647 (53.79) |
| ≥65 years | 3,535,090 (32.35) | 4179006 (36.02) | 4155042 (35.79) | 4,172,390 (35.84) |
| Sex, n (%) |  |  |  |  |
| Female | 5,635,709 (51.57) | 6,008,586 (51.78) | 6,014,816 (51.80) | 6,032,695 (51.82) |
| Male | 5,291,644 (48.43) | 5,594,406 (48.22) | 5,596,138 (48.20) | 5,608,028 (48.18) |
| Race and ethnicity, n (%) |  |  |  |  |
| Asian | 595,356 (5.45) | 622,155 (5.36) | 626,293 (5.39) | 627,281 (5.39) |
| Black | 1,082,443 (9.91) | 1,155,319 (9.96) | 1,157,239 (9.97) | 1,152,748 (9.90) |
| Hispanic | 1,339,113 (12.25) | 1,417,443 (12.22) | 1,424,074 (12.26) | 1,425,600 (12.25) |
| Other | 422,523 (3.87) | 454,749 (3.92) | 457,557 (3.94) | 469,861 (4.04) |
| White | 7,487,918 (68.52) | 7,953,326 (68.55) | 7,945,791 (68.43) | 7,965,233 (68.43) |
