# Supplementary material for: Trends in the incidence of asthma, atopic dermatitis, and multiple sclerosis before, during, and after the COVID-19 pandemic in a US claims database
Source: PLoS One. 2026 Jul 30;21(7):e0355103. doi: 10.1371/journal.pone.0355103 (PMC13422859; doi:10.1371/journal.pone.0355103)
Supplement: S1 Fig — Standardized IRs were calculated using a direct method with the population composition by age and sex in the United States in 2022 as the reference population. (DOCX) [file pone.0355103.s003.docx]

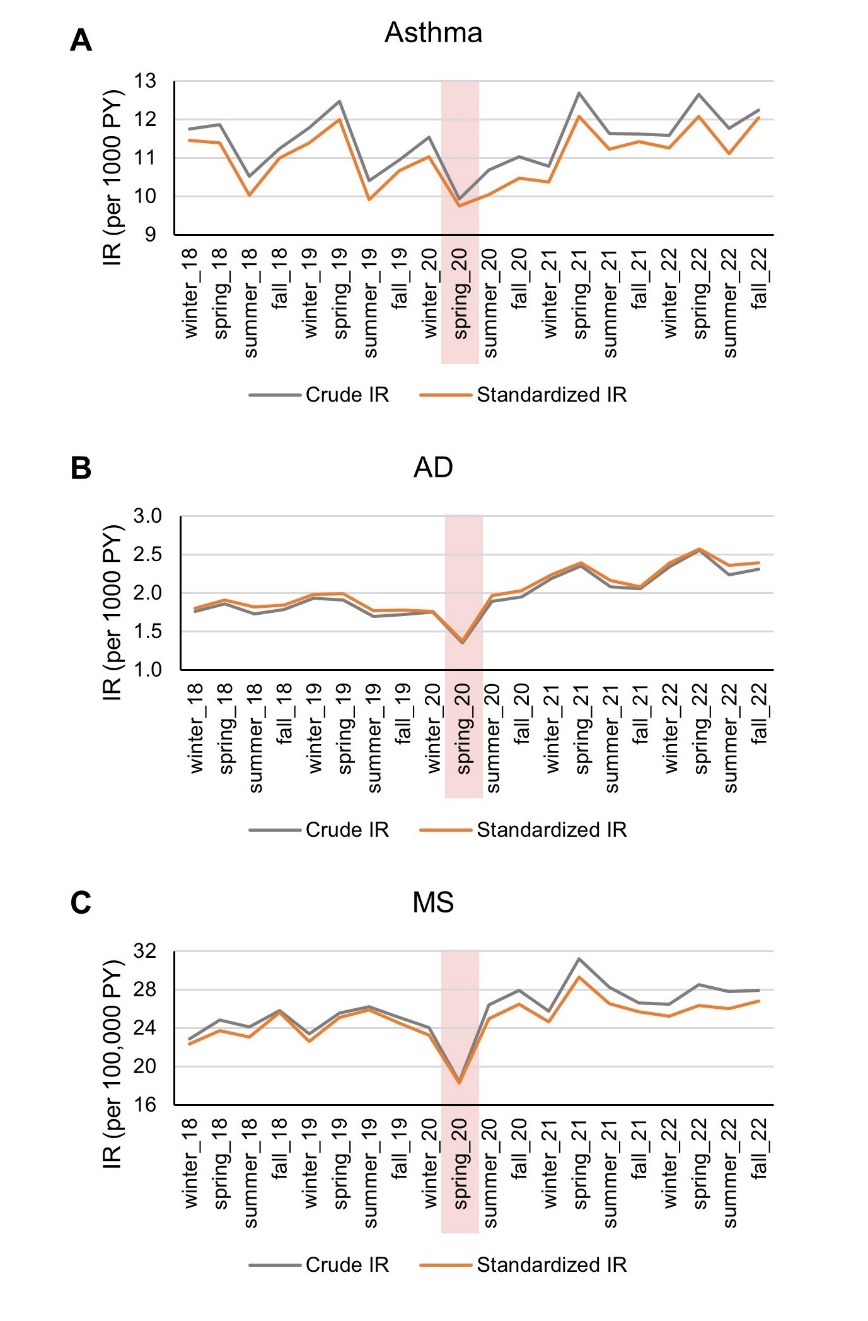


# S1 Fig. Incidence rates (IR) of asthma, atopic dermatitis (AD), and multiple sclerosis (MS) in seasonal cohorts from 2018 to 2022 in Optum^®^ CDM.

Standardized IRs were calculated using a direct method with the population composition by age and sex in the United States in 2022 as the reference population.
