# Supplementary material for: Trends in the incidence of asthma, atopic dermatitis, and multiple sclerosis before, during, and after the COVID-19 pandemic in a US claims database
Source: PLoS One. 2026 Jul 30;21(7):e0355103. doi: 10.1371/journal.pone.0355103 (PMC13422859; doi:10.1371/journal.pone.0355103)
Supplement: S2 Fig — Panels A-B, D-E, and G-H: IRs for asthma, AD, and MS were calculated by age and sex from 2018 to 2022. Panels C, F, and I: IRRs were calculated to compare the IRs in age and sex subgroups in spring 2020 to the IRs in the corresponding age and sex subgroups in spring 2018. Dots represent the IRRs and error bars represent the 95% confidence intervals. Dash lines represent an IRR of 1. (DOCX) [file pone.0355103.s004.docx]

**
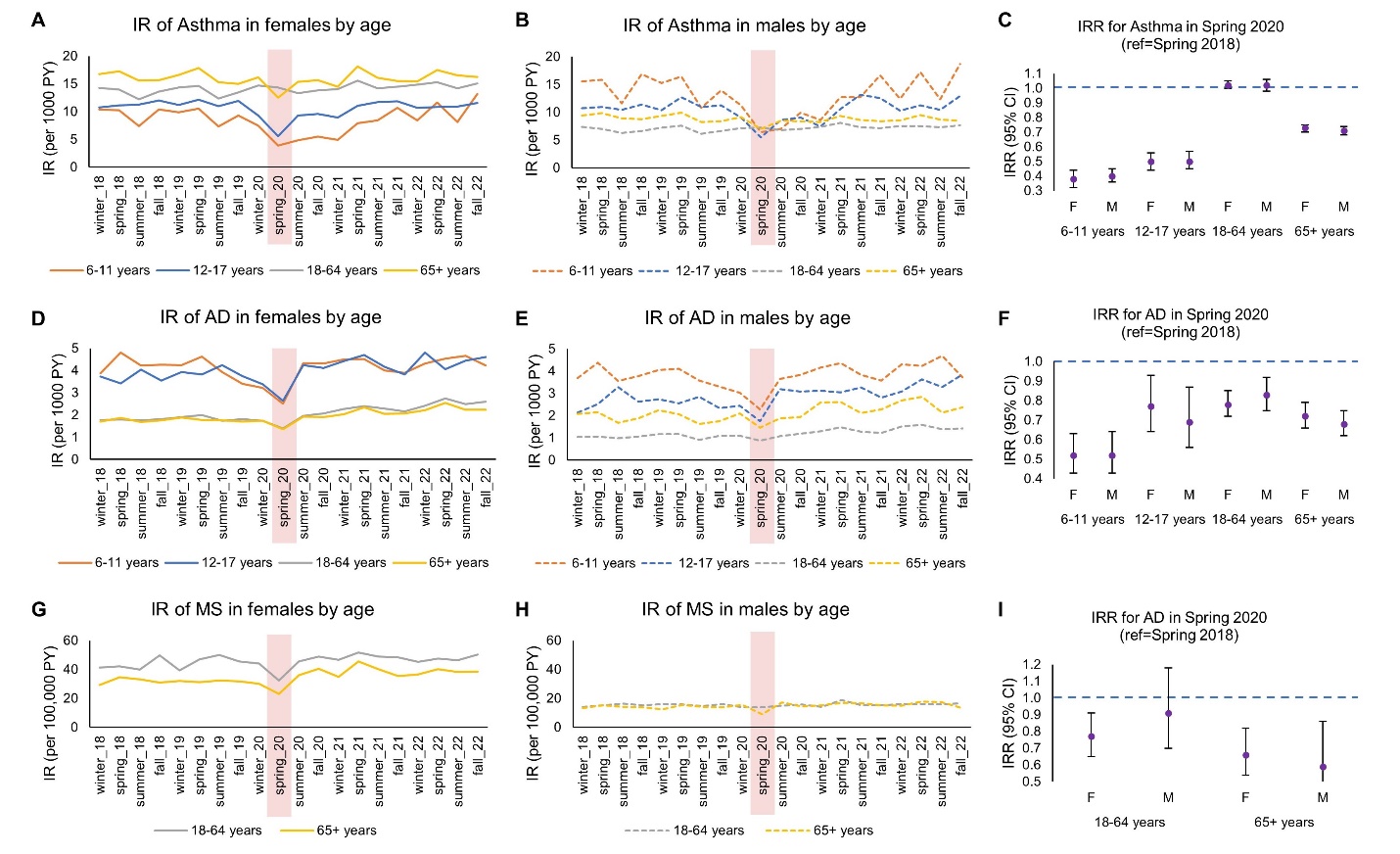
**

# S2 Fig. Incidence rates (IR) and incidence rate ratios (IRR) of asthma, atopic dermatitis (AD), and multiple sclerosis (MS) in seasonal cohorts from 2018 to 2022 by age and sex in Optum^®^ CDM.

Panels A-B, D-E, and G-H: IRs for asthma, AD, and MS were calculated by age and sex from 2018 to 2022. Panels C, F, and I: IRRs were calculated to compare the IRs in age and sex subgroups in spring 2020 to the IRs in the corresponding age and sex subgroups in spring 2018. Dots represent the IRRs and error bars represent the 95% confidence intervals. Dash lines represent an IRR of 1.
